# Utilisation of Mucin Glycans by the Human Gut Symbiont *Ruminococcus gnavus* Is Strain-Dependent

**DOI:** 10.1371/journal.pone.0076341

**Published:** 2013-10-25

**Authors:** Emmanuelle H. Crost, Louise E. Tailford, Gwenaelle Le Gall, Michel Fons, Bernard Henrissat, Nathalie Juge

**Affiliations:** 1 The Gut Health and Food Safety Institute Strategic Programme, Institute of Food Research, Norwich, United Kingdom; 2 Laboratoire de Chimie Bactérienne, Institut de Microbiologie de la Méditerranée, CNRS and Aix-Marseille University, Marseille, France; 3 Architecture et Fonction des Macromolécules Biologiques, CNRS and Aix-Marseille University, Marseille, France; 4 Department of Cellular and Molecular Medicine, Faculty of Health and Medical Sciences, University of Copenhagen, Copenhagen, Denmark; University of Florida, United States of America

## Abstract

Commensal bacteria often have an especially rich source of glycan-degrading enzymes which allow them to utilize undigested carbohydrates from the food or the host. The species *Ruminococcus gnavus* is present in the digestive tract of ≥90% of humans and has been implicated in gut-related diseases such as inflammatory bowel diseases (IBD). Here we analysed the ability of two *R. gnavus* human strains, E1 and ATCC 29149, to utilize host glycans. We showed that although both strains could assimilate mucin monosaccharides, only *R. gnavus* ATCC 29149 was able to grow on mucin as a sole carbon source. Comparative genomic analysis of the two *R. gnavus* strains highlighted potential clusters and glycoside hydrolases (GHs) responsible for the breakdown and utilization of mucin-derived glycans. Transcriptomic and functional activity assays confirmed the importance of specific GH33 sialidase, and GH29 and GH95 fucosidases in the mucin utilisation pathway. Notably, we uncovered a novel pathway by which *R. gnavus* ATCC 29149 utilises sialic acid from sialylated substrates. Our results also demonstrated the ability of *R. gnavus* ATCC 29149 to produce propanol and propionate as the end products of metabolism when grown on mucin and fucosylated glycans. These new findings provide molecular insights into the strain-specificity of *R. gnavus* adaptation to the gut environment advancing our understanding of the role of gut commensals in health and disease.

## Introduction

The human gastrointestinal (GI) tract contains a dynamic community of trillions of microorganisms leaving in a symbiotic relationship with the host [1]. Two phyla, Bacteroidetes and Firmicutes, dominate gut microbiota biodiversity [2], [3]. These symbionts have adapted to maximise metabolic access to a wide variety of dietary- and host-derived carbohydrates (mucin glycans), and competition for these nutrients is considered as a major factor shaping the structure-function of the microbiota [4]. The gut microbiota provides many crucial functions to the host including calorie extraction from the diet, generation of short-chain fatty acids (SCFAs), metabolism of xenobiotics, development of immune system and pathogen exclusion [5], [6]. In healthy subjects, the composition of the adult gut microbiota is remarkably stable [7]. In contrast, deviation away from gut microbial balance, or ‘dysbiosis’, has been repeatedly reported in diseases such as inflammatory bowel diseases (IBD) including ulcerative colitis (UC) and Crohn's disease (CD) [8]. Some changes in the microbial community are shared in CD and UC including reduced biodiversity (in particular Firmicutes), temporal instability and increased mucosa-associated bacteria [9], [10], [11].

The epithelial cells of the mammalian intestine are covered with a mucus layer that prevents direct contact with intestinal microbes but also constitutes a substrate for mucus-adapted bacteria [12]. Mucins are *O*-linked *N*-acetylgalactosamine (GalNAc) glycoproteins, constituting the major structural components of mucus [13]. The *O*-glycan structures present in mucin are diverse and complex and consist predominantly of core 1–4 mucin-type O-glycans containing GalNAc, galactose (Gal) and N-acetyl-glucosamine (GlcNAc) [14]. Gastric and duodenal mucins generally contain the core-1 (Galβ1–3GalNAcα1-Ser/Thr) and the core-2 (Galβ1–3(GlcNAcβ1–6)GalNAcα1-Ser/Thr) structures. Recent studies revealed that MUC2 in the sigmoid colon mainly contains the core-3 structure (GlcNAcβ1-3GalNAcα1-Ser/Thr) [15]. These core structures are further elongated and frequently modified by fucose and sialic acid residues via α1-2/3/4 and α2-3/6 linkages, respectively. The proportion of sialic acid in human intestinal mucin increases proportionally from the ileum to the rectum [16]. Microbial communities that are strongly associated with the mucosa are different from those that are frequently sampled from the faeces, with an overrepresentation of bacteria that degrade mucins [17], [18], [19], [20]. Given the diversity and complexity of mucin structures found within the gut, strategies for deconstructing these molecules rely on the cooperative action of a number of carbohydrate-active enzymes (CAZymes) encoded by the genome of mucin-using bacteria [21]. The ability of certain microorganisms to utilize these endogenous glycans may thus facilitate their close location to the host cells where they may exert a disproportionate effect on human health, especially during states of dysbiosis [22].

*Ruminococcus gnavus* is a Gram-positive anaerobic bacterium, belonging to the Firmicutes division, *Clostridia* class and XIVa cluster, Lachnospiraceae family [23]. A recent molecular inventory revealed that *R. gnavus* is widely distributed amongst individuals, and is represented in the most common 57 species present in ≥90% of individuals [24]. Colonisation by *R. gnavus* was found in infants during the first days of life [25]. *R. gnavus* is in the top 15 species showing abundance in both adult and infant gut-enriched genes, supporting *R. gnavus* adaptation to the intestinal habitat throughout life [26]. Among Firmicutes, *R. gnavus* appears to be particularly over-represented in CD patients. Comparison between ileal mucosa samples of healthy individuals with patients suffering from ileal CD revealed an increased abundance of *R. gnavus* with a reduced abundance of *Faecalibacterium prausnitzii* in the CD patients [27]. The same findings were observed in faecal samples from CD patients compared to unaffected controls [28]. An earlier study reported that colonic biopsies from CD-afflicted patients compared with biopsies from normal control subjects had an increase in anaerobic bacteria; in small bowel, CD patients had an increase in the *R. gnavus* subgroup with a decrease in the *Clostridium leptum* and *Prevotella nigrescens* subgroups [29]. Furthermore *R. gnavus* was increased in macroscocopically and histologically normal intestinal epithelium of both CD and UC patients [30]. A different pattern was observed in patients with active UC, where *R. gnavus* was found abundantly present in the colonic mucosa of healthy subjects but lost during active UC [31]. These studies point towards an important role of *R. gnavus* in modulating gut inflammatory response at the mucosal surface.

Here we investigated the ability of *R. gnavus* strains to utilise mucins, providing molecular insights into features that determine bacteria adaptation to the gut mucosal environment in health and disease.

## Materials and Methods

### Materials

All the monosaccharides, D-glucose (Glc), D-galactose (Gal), N-acetyl-D-glucosamine (GlcNAc), N-acetyl-D-galactosamine (GalNAc), L-fucose (Fuc), D-lactose (Lac), N-acetylneuraminic acid (Neu5Ac), N-glycolylneuraminic acid (Neu5Gc) as well as 2′-(4-Methylumbelliferyl)- α-D-*N*-acetylneuraminic acid (4MU-Neu5Ac) and type III pig gastric mucin (PGM) were purchased from Sigma-Aldrich (St Louis, MO). Purified pig gastric mucin (pPGM) was obtained as previously described [32]. The oligosaccharides, 2′-fucosyllactose (2′FL), 3-fucosyllactose (3FL), lacto-N-neo-tetraose (LNnT) lacto-N-tetraose (LNT) and 6′-O-sialyllactose (6′SL) were kindly provided by Glycom A/S (Lyngby, Denmark). 3′-sialyllactose (3′SL) and N-acetyl-D-lactosamine (LacNAc) were purchased from Carbosynth Limited (Campton, UK).

### Bacterial strains and growth conditions

The E1 strain has been isolated from the predominant faecal microbiota of a healthy human adult [33] and further identified as *R. gnavus* [34]. *R. gnavus* ATCC 29149, originally designated as *Ruminococcus* AB, has also been isolated from fecal sample of a healthy human adult [35].

*R. gnavus* strains were routinely grown in an anaerobic cabinet (Don Whitley, Shipley, UK) in brain heart infusion broth supplemented with yeast extract and hemin [BHI-YH; BHI (Oxoid LTD, Basingstoke, UK) supplemented with 5 g.L^−1^ of Bacto™ yeast extract (Becton, Dickinson and Company, Sparks, MD) and 5 mg.L^−1^ of hemin (Sigma-Aldrich)]. Growth on single-carbon sources utilized anaerobic basal YCFA medium supplemented with 27.7 mM of specific mono- or oligosaccharides as indicated or 1% (wt/vol) of purified pig gastric mucin. YCFA medium consisted of (per 1 L): 10 g casitone, 2.5 g yeast extract, 4 g NaHCO_3_, 1 g L-cysteine hydrochloride, 450 mg K_2_HPO_4_, 450 mg KH_2_PO_4_, 900 mg NaCl, 90 mg MgSO_4_.7H_2_O, 90 mg CaCl_2_, 1 mg resazurin, 10 mg hemin, 10 µg biotin, 10 µg cobalamin, 30 µg *p*-aminobenzoic acid, 50 µg folic acid and 150 µg pyridoxamine [36]. Note that YCFA medium usually contain (NH4)2SO4 as later described [37]. Final concentrations of short-chain fatty acids (SCFA) in the medium were 33 mM acetate, 9 mM propionate and 1 mM each of isobutyrate, isovalerate and valerate. The pH was adjusted to 6.5. The medium was prepared under a headspace of 85% N_2_, 10% H_2_ and 5% CO_2_ gas mix. Thiamine and riboflavin were added anaerobically to the medium to give a final concentration of 50 µg.L^−1^ each and then the medium was autoclaved. Growth was determined spectrophotometrically by monitoring changes in optical density at 600 nm compared to the same medium without bacterium (OD600 nm). The in-house-developed DMFit program (http://www.combase.cc/index.php/en/downloads/file/53-dmfit-30) was used with the scale-free option to compare the effect of the carbon source on growth rates [38].

### Comparative CAZome analysis

The translated protein sequences of *R. gnavus* ATCC 29149 and *R. gnavus* E1 were compared to the full length sequences derived from the Carbohydrate-Active enZymes (CAZy) database (www.cazy.org; [39]) using BLAST [40]. The sequences that had an e-value >0.1 were assigned to GH, GT, PL, CE and CBM families using a parallel procedure involving a BLAST search against partial sequences corresponding to individual GH, GT, PL, CE and CBM modules and a HMMer search [41] using hidden Markov models built for each CAZy module family[39]. The counts for each CAZy family of each strain were then compared and the putative function of the proteins of interest was evaluated by alignment with the sequences of biochemically characterized enzymes [39].

### Total RNA extraction from *R. gnavus* ATCC 29149

Total RNA was extracted from 3 mL of mid- to late exponential phase cultures of ATCC 29149 in YCFA supplemented with one carbon source (Glc, GalFuc, 2′FL, 3FL, 3′SL or pPGM). Two biological replicates were performed for each carbon source except Glc. The RNA was stabilized prior to extraction by using RNAprotect Bacteria Reagent (Qiagen, Crawley, UK) according to supplier's advice. The RNA was then extracted after an enzymatic lysis followed by a mechanical discruption of the cells, using the RNeasy Mini Kit (Qiagen) according to manufacturer's instructions. Genomic DNA contamination was removed by DNAse treatment using TURBO DNA-free kit (Life Technologies Ltd, Paisley, UK) according to supplier's recommendations. The purity, quantity and integrity of the extracted RNA were assessed before and after DNAse treatment, with NanoDrop 1000 UV-Vis Spectrophotometer (Thermo Fischer Scientific, Wilmington, DE) and with Agilent RNA 600 Nano kit on Agilent 2100 Bioanalyzer (Agilent Technologies, Stockport, UK).

### Genomic DNA extraction from *R. gnavus* ATCC 29149

For the isolation of *R. gnavus* ATCC 29149-chromosomal DNA, cells from a 50 mL-overnight culture were harvested by centrifugation (10,000 g, 5 min, 4°C). The cell pellet was washed with 5 mL of TES buffer (10 mM Tris, 1 mM EDTA, 0.1 M NaCl, pH8), resuspended in 5 mL of TES buffer supplemented with lysozyme (20 mg.mL^−1^) and incubated for 15 min at 37°C. Then, complete lysis was achieved by addition of 1 mL of 20% sodium dodecyl sulfate (SDS) and incubation for 10 min at 50°C. The mixture was then extracted by three consecutive treatments: first, with 5 mL of phenol pH 7.9 then with 5 mL of phenol-chloroform-isoamyl alcohol (25∶24∶1) and finally with 5 mL of chloroform-isoamyl alcohol (24∶1). After precipitation with cold absolute ethanol, the genomic DNA was resuspended in 2 mL of TE buffer (10 mM Tris, 1 mM EDTA, pH8). Traces of RNA were removed by a treatment with RNAse ONE (Promega, Madison, WI) used as recommended by the manufacturer. The DNA was again precipitated with 0.3 M sodium acetate (pH5.2) and 70% ice-cold ethanol. Finally, it was dissolved in 1.5 mL of TE. Quality and quantity were assessed using NanoDrop 1000 UV-Vis Spectrophotometer.

### Transcriptional profiling by microarray

A total of 1499 60-mer probes were designed for microarray experiments based on *R. gnavus* ATCC 29149 genome information using Array Designer 3.0 software (PREMIER Biosoft International, Palo Alto, CA) and printed on Agilent Custom Oligonucleotide Microarrays 8×15 k. For sample preparation, the Sau3AI-digested ATCC 29149 genomic DNA (gDNA) and each cDNA were fluorescently labelled using the BioPrime® Array CGH Genomic Labeling System (Life Technologies Ltd) according to supplier's instructions, and Cy3-dUTP or Cy5-dUTP respectively (GE Healthcare UK Ltd, Little Chalfont, UK). The microarrays were then hybridized overnight at 63°C with Cy5-cDNA/Cy3-gDNA mixtures prepared according to supplier's advice. The slides were scanned on GenePix® 4000B scanner (Molecular Devices, Inc., Sunnyvale, CA). Image processing was done with GenePix Pro 6.0 software (Molecular Devices, Inc.). Data analysis was performed using GeneSpringGX version 7.3 software (Agilent Technologies). A per spot and per chip intensity-dependent normalization (also called LOWESS normalization) was applied using corrected signal obtained for Cy3-gDNA at 532 nm as a control signal (see Protocol S1 for detailed information).

### Quantitative real-time PCR (qPCR)

qPCR was carried out in an Applied Biosystems 7500 Real-Time PCR system (Life Technologies Ltd). One pair of primers was designed for each target gene using ProbeFinder version 2.45 (Roche Applied Science, Penzberg, Germany) to obtain an amplicon of around 60–80 bp long. The primers were between 18 and 23 nt-long, with a T_m_ of 59–60°C (Table S1). Calibration curves were prepared in triplicates for each pair of primers using 2.5-fold serial dilutions of *R. gnavus* ATCC 29149 genomic DNA. The standard curves showed a linear relationship of log input DNA vs. the threshold cycle (C_T_), with acceptable values for the slopes and the regression coefficients (R^2^). The dissociation curves were also performed to check the specificity of the amplicons. Each DNAse-treated RNA (1 µg) was converted into cDNA using QuantiTect® Reverse Transcription kit (Qiagen) according to supplier's advice. DNAse-treated RNA was also treated the same way but without addition of the reverse-transcriptase (RT−). Each qPCR reaction (10 µL) was then carried out in triplicates with 1 µL of a 20-fold diluted sample (cDNA or RT−) and 0.2 µM of each primer, using the QuantiFast SYBR Green PCR kit (Qiagen) according to supplier's advice (except that the combined annealing/extension step was extended to 35 s instead of 30 s).

Data obtained with cDNA were analyzed only when C_T_ values above 36 were obtained for the corresponding RT−. For each cDNA sample, the 3 C_T_ values obtained for each gene were averaged. The data were then analyzed using the 2^−ΔΔCT^ method using housekeeping *gyrB* (RUMGNA_00867) gene as a reference gene and glucose as a reference condition. For each gene in each condition, the final value of the relative level of transcription (expressed as a fold change in gene transcription compared to glucose) is an average of 2 biological replicates. Data were analysed using 1-way ANOVA. A post-hoc test (Dunnett's) was used to examine if there were any significant differences in each treatment (versus the control treatment).

### ^1^H nuclear magnetic resonance analysis

^1^H NMR was used to identify the presence, absence, and concentration of several metabolites in *R. gnavus* growth medium. Supernatant samples were thawed at room temperature and prepared for ^1^H NMR spectroscopy by mixing 400 µL of spent medium with 200 µL of phosphate buffer (0.2MNa2HPO4, 0.038 M NaH2PO4 [pH 7.4]) made up in 100% D_2_O and containing 0.06% sodium azide, and 1.5 mM DSS (sodium 2,2-dimethyl-2-silapentane- 5-sulfonate) as a chemical shift reference. The sample was mixed, and 500 µL was transferred into a 5-mm NMR tube for spectral acquisition. The ^1^H NMR spectra were recorded at 600 MHz on a Bruker Avance spectrometer (Bruker BioSpin GmbH, Rheinstetten, Germany) running Topspin 2.0 software and fitted with a cryoprobe and a 60-slot autosampler. Each ^1^H NMR spectrum was acquired with 128 scans, a spectral width of 8,012.8 Hz, an acquisition time of 2.04 s, and a relaxation delay of 2.0 s. The “noesypr1d” presaturation sequence was used to suppress the residual water signal with a low-power selective irradiation at the water frequency during the recycle delay and a mixing time of 100 ms. Spectra were transformed with a 0.3-Hz line broadening, manually phased, baseline corrected, and referenced by setting the DSS methyl signal to 0 ppm.

### Enzymatic assays

Sialidase activities of *R. gnavus* ATCC 29149 and E1 were examined as follows. *R. gnavus* strains were inoculated into 5 mL of YCFA broth supplemented with a single carbon source for up to 28 h under anaerobic conditions (as described above). The cell density was monitored at OD600 nm and 1 mL aliquots removed from the culture at 6, 9 and 28 h. The cells were removed by centrifugation (17,000 g, 5 min, 4°C). The supernatant was stored at −20°C until required. For the enzymatic assay, the supernatant (at 1/5 total reaction volume) was added to a reaction mixture consisting of 500 µM 4MU-Neu5Ac as a substrate in PBS pH 7.4. The enzymatic reactions were carried out at 37°C for up to 2 h in an incubated platereader (BMG Labtech, Ortenberg, Germany). The fluorescence of the liberated 4MU was quantified at Excitation 340 and Emission 420 nm automatically at 5 min intervals in the plate reader. The rate of MU release/min was calculated using data from the linear portion (∼20–40 min) of the reaction using Prism 6 (GraphPad Software CA, USA), and corrected by subtracting the “No enzyme” control rates. This rate was then divided by the OD600 for the cell culture at this time. ^1^H NMR was used to analyze the reaction products. For this, an appropriate amount of *R. gnavus* ATCC 29149 supernatant (1/5 to 1/10 reaction volume) was incubated with the following substrates in PBS pH 7.4 at 37°C: 3′SL (1.5 mM); 4MU-Neu5Ac (0.5 mM) for 2 h to 24 h. The reaction was stopped by denaturing the enzyme by boiling for 20 min, the denatured enzyme and any particulate material was removed by centrifugation at 17 000 g, 4°C for 10 min, and the supernatant was analyzed by ^1^H NMR (see above) by mixing 400 µL of medium with 200 µL of D_2_O and 20 µL of a solution of 1 mM d_4_-TSP (sodium 3-(trimethylsilyl)-propionate-d_4_).

### Nucleotide and protein sequence analyses

Protein sequence homologies were searched using the blastp software (http://blast.ncbi.nlm.nih.gov/Blast.cgi). SignalP 4.1 server (http://www.cbs.dtu.dk/services/SignalP/) and CW-PRED software (http://bioinformatics.biol.uoa.gr/CW-PRED/) were used to predict signal peptides and cell-wall anchored proteins, respectively. These analyses were completed by a prediction of the cellular localisation of the protein using PSORTb version 3.0.2 (http://www.psort.org/psortb/). Putative transcriptional terminators were predicted in silico using the RNAfold program (http://rna.tbi.univie.ac.at/cgi-bin/RNAfold.cgi). Prediction of the promoters was performed using the BPROM program (http://linux1.softberry.com/berry.phtml?topic=bprom&group=programs&subgroup=gfindb).

## Results

### Comparative analysis of *R. gnavus* E1 and *R. gnavus* ATCC 29149 glycobiome

**Figure 1:**
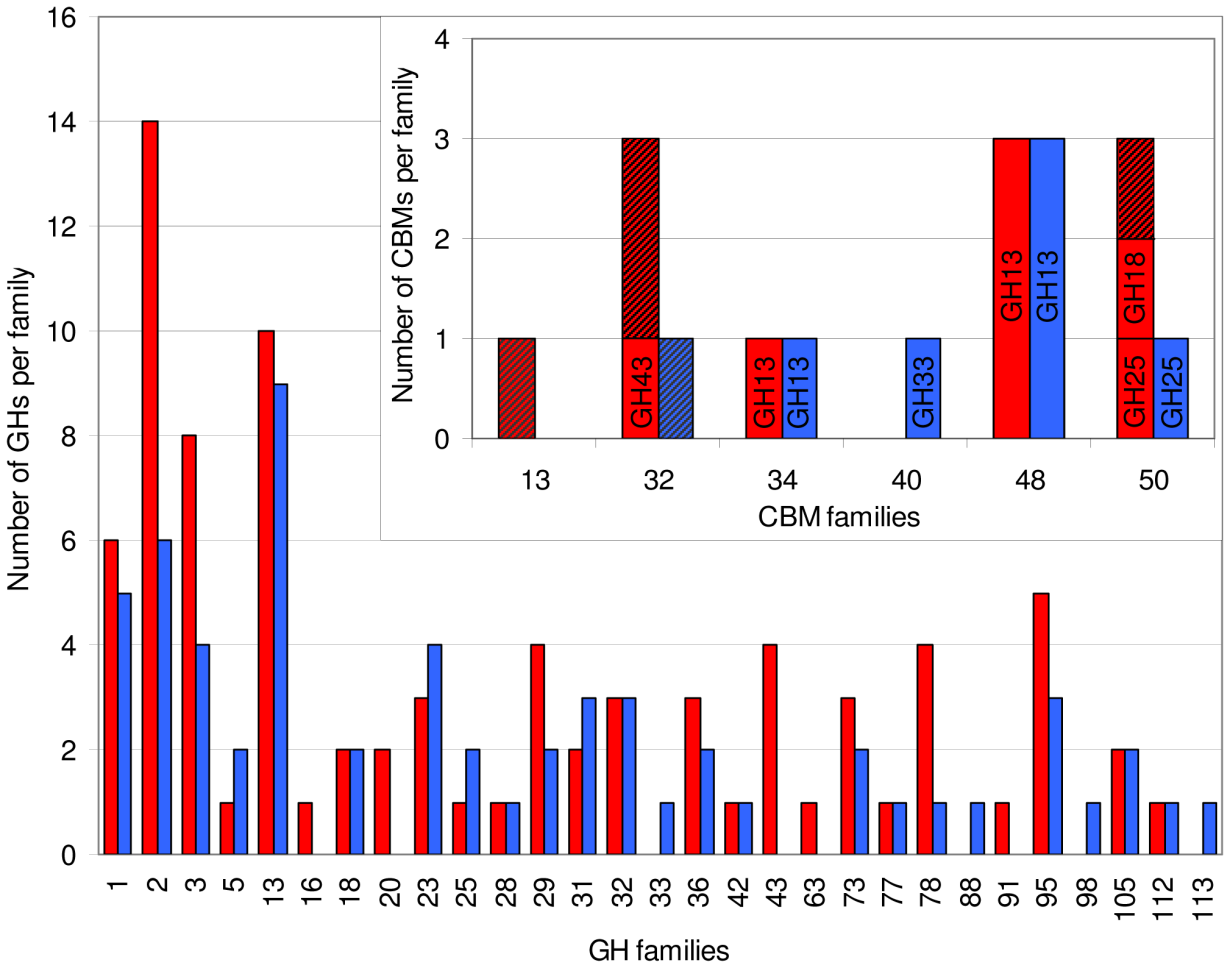
Comparison of the distribution of GHs and CBM between *R. gnavus* E1 and ATCC 29149. GHs and CBMs are represented by red boxes for *R. gnavus* E1 and by blue boxes for *R. gnavus* ATCC 29149. CBMs associated with GH are represented by plain boxes, with the GH family indicated inside the box. CBMs not associated with GH are represented by striped boxes.

The genome of *R. gnavus* E1 was recently sequenced (Genoscope, Evry, France); genomic analysis identified 112 full length and 5 fragments of genes encoding CAZymes (www.cazy.org) [39], corresponding to approximately 3.7% of genes dedicated to carbohydrate metabolism. *R. gnavus* E1 CAZome contains 23 glycoside transferases (GT), 6 carbohydrate esterases (CE), 11 carbohydrate binding module (CBM) and 84 GHs. Most of *R. gnavus* E1 CAZome is represented by genes encoding GHs distributed into 25 GH families. The most represented are the GH2 (16.7%), GH13 (11.9%), GH3 (9.5%) and GH1 (7.1%) families which mostly contain enzymes generally active on plant-derived substrates. The larger *R. gnavus* ATCC 29149 genome displays 60 predicted GHs across 24 GH families. A comparison of *R. gnavus* E1 GH and CBM repertoire with that of *R. gnavus* ATCC 29149 strain is presented in Fig. 1. Both strains possess similar number of GH13 enzymes while the E1 strain has a higher number of GH1, GH2 and GH3, thus, together with a higher number of GH36 (α-galactosidase), GH78 (rhamnosidase), GH43 (xylosidase/arabinosidase), GH29 and GH95 (α-fucosidases), and strain-specific GH63 (α-glucosidase), GH16 (β-glucanase), GH91 (inulin fructotransferase), the E1 strain seems to be more adapted to the degradation of a diversified array of dietary carbohydrate-based substrates [42]. In contrast, the *R. gnavus* ATCC 29149 genome encodes less GHs than E1 but with a higher proportion of enzymes putatively implicated in degradation of host-derived oligosaccharides, including predicted GH33 sialidase and GH98 endo-β-galactosidase, which are absent in the *R. gnavus* E1 genome, and both predicted to be extracellular. CBMs that recognize mammalian glycans presently belong to relatively few CBM families – families 32, 40, 41, 47, and 51 [43]. CBM32s are found in both *R. gnavus* E1 and ATCC 29149 strains whereas CBM40 is specific to ATCC 29149 (Fig. 1). At present CBMs in family 40 are the only known examples to bind sialic acid and are exclusively associated with sialidases [43]. A CBM40 is associated with the putative GH33 sialidase in *R. gnavus* ATCC 29149, possibly enhancing the ability of the enzyme to attach and degrade mucins. Moreover, the genomes of both *R. gnavus* E1 and ATCC 29149 encode many GH29 and GH95 fucosidases which may play a role in the degradation of host and/or dietary glycans. Apart from this glycolytic potential, the molecular basis for transmembrane import of oligosaccharides is evident from various ATP-binding cassette transporters and PTS (not shown).

### *R. gnavus* E1 and *R. gnavus* ATCC 29149 strains differentially consume mucin

**Figure 2:**
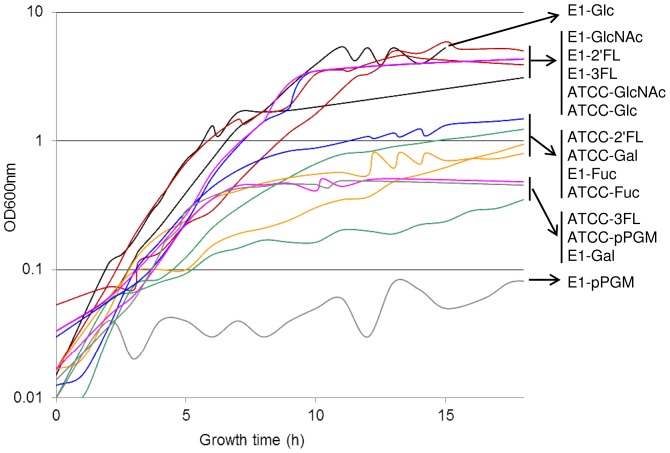
Growth curves of *R. gnavus* E1 and ATCC29149 with different carbohydrates as sole carbon source. For each sugar and each strain, the growth curve represent the average growth, measured at OD600(Black, Glc; Red, GlcNAc; Green, Gal; Orange, Fuc; Blue, 2′FL and Pink, 3FL).

We first monitored the anaerobic growth of *R. gnavus* E1 and *R. gnavus* ATCC 29149 on basal medium supplemented with diverse monosaccharides and host oligosaccharides as carbon sources (Fig. 2 and Table 1). Spectrophotometric measurements were made every hour for up to 40 h, and the growth curves analyzed using the in-house-developed DMFit program, enabling quantitative measurements of both growth rate and final culture density for each sugar (Table 1). Both *R. gnavus* E1 and *R. gnavus* ATCC 29149 grew on monosaccharides Glc, Gal, Fuc, GlcNAc as substrates whereas the strains were unable to grow in presence of GalNAc or sialic acid (Neu5Ac or Neu5Gc) as sole carbon source (Table 1). The lack of growth of these strains on sialic acid is surprising as the *R. gnavus* ATCC 29149 genome possesses the complete cluster of genes (the *nan* cluster) encoding proteins necessary for the catabolism of sialic acid including putative transporters (see below). Interestingly the *R. gnavus* strains were able to grow on GlcNAc but not on GalNAc as sole carbon source; in enteric bacteria, the aminosugars are transported by specific PTSs and enter the aminosugar metabolic cycle after phosphorylation, *via* the Leloir-like pathway consisting of common enzymes identified in *Bibidobacterium bifidum* [44]. The *nagE* gene encoding GlcNAc specific PTS (PTSII^Nag^) is present in both E1 (RUGNEv3_10975) and ATCC 29149 (RUMGNA_03053) whereas the GalNAc specific PTS is only present in ATCC 29149 containing the IIA (RUMGNA_00960), IIB (RUMGNA_00962), IIC (RUMGNA_00963) and IID (RUMGNA_00964) components. Only *R. gnavus* E1 was able to grow on Lac (Galβ1-4Glc) as sole carbon source. β-galactosidase activity (catalysing Lac hydrolysis) can be found in GH1, 2, 35 and 42 families [45]. Homology searches suggest that, in *R. gnavus* E1, β-galactosidases are predicted in GH2 (RUGNEv3_10547, 10622, 50063, 50166, 60208, 60218 and 61117) and GH42 (RUGNEv3_10179), and more surprisingly in GH43 (RUGNEv3_10174) families whereas they are either absent or showing low identity with homologues in ATCC 29149 and thus represent good candidates to explain the differences in Lac utilisation between the two *R. gnavus* strains.

**Table 1:** Growth rate and density of *R. gnavus* E1 and ATCC 29149 growth supplemented with different carbohydrates.

	E1			ATCC		
Sugar:	Growth	Relative growth rate*	ΔODmax	Growth	Relative growth rate*	ΔODmax
Glc *(Positive control)*	+	0.87	4.81	+	1.00	4.00
GlcNAc	+	0.63	5.41	+	0.92	3.73
Gal	+	0.17	3.50	+	0.53	2.00
GalNAc	−	n/a	n/a	−	n/a	n/a
Fuc	+	0.25	2.21	+	0.72	2.10
Neu5Ac	−	n/a	n/a	−	n/a	n/a
Neu5Gc	nt	n/a	n/a	−	n/a	n/a
Lac	+	nd	nd	−	n/a	n/a
2′FL	+	0.84	5.32	+	0.86	1.57
3FL	+	0.81	5.06	+	0.80	0.49
LNT	−	n/a	n/a	−	n/a	n/a
LNnT	−	n/a	n/a	−	n/a	n/a
LacNAc	+	nd	nd	−	n/a	n/a
6′SL	−	n/a	n/a	−	n/a	n/a
3′SL	−	n/a	n/a	+	nd	nd
pPGM	−	n/a	n/a	+	1.18	0.44

− = no growth; + = growth; nt = not tested; nd = not determined; n/a = not applicable.

* All growth rates are expressed as a relative value to the growth rate of ATCC 29149 with Glc.

**Figure 3:**
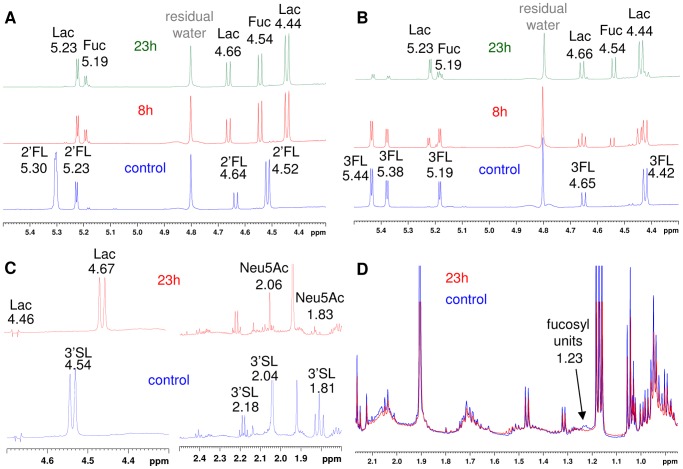
H^1^ NMR analysis of *R. gnavus* ATCC 29149 culture supernatant. YCFA medium supplemented with2′FL (A), 3FL (B), 3′SL (C) or pPGM (D) were analysed by H^1^ NMR before (control) or after 8 h or 23 h of growth of *R. gnavus* ATCC 29149 to assess substrate utilization. Peaks were assigned by using the appropriate sugar standards and based on literature.

Both *R. gnavus* E1 and *R. gnavus* ATCC 29149 grew on 2′-fucosyllactose (Fucα1,2Galβ1,4Glc, 2′FL) and 3-fucosyllactose (Galβ-4[Fucα-3]Glc, 3FL) (Fig. 2 and Table 1) but not on type 1 Lacto-N-tetraose (Galβ1-3GlcNAcβ13Galβ1-4Glc, LNT) or type-2 Lacto-N-neo-tetraose (Galβ1-4GlcNAcβ13Galβ1-4Glc, LNnT) human milk oligosaccharides (HMOs). ^1^H NMR experiments showed that *R. gnavus* growth on 2′FL and 3FL coincides with the release of Fuc from these substrates rather than transport of the fucosylated oligosaccharides and assimilation inside the cells (Fig. 3A/B), in agreement with the presence of predicted extracellular GH29 and GH95 fucosidases in both *R. gnavus* strains. In characterized HMO-degrading bifidobacteria strains, type-2 HMOs are sequentially degraded by GH2 β-galactosidases, acting on LacNAc and GH20 β-*N*-acetylhexosaminidases, specific for GlcNAcβ1–3Galβ1-R [46] whereas degradation of type-1 chains relies on expression of GH20 lacto-*N*-biosidase which is required for the release of lacto-N-biose I (Galβ1-3GlcNAc, LNB) from the tetrasaccharide [47]. Since Gal and GlcNAc are good substrates of these strains, the lack of growth of *R. gnavus* E1 and ATCC 29149 on LNnT suggests that *R. gnavus* lacks the enzymatic specificity required for the release of Gal or GlcNAc from the tetrasaccharide, despite the presence of 14 and 6 predicted GH2 β-galactosidases in *R. gnavus* E1 and ATCC 29149, respectively and two putative GH20 β-*N*-acetylhexosaminidases in *R. gnavus* E1. In addition, since *R. gnavus* E1, but not ATCC 29149, was able to grow on *N*-acetyllactosamine (Galβ1-4GlcNAc, LacNAc) (Fig. 2 and Table 1), these experiments suggest that no LacNAc could be released from the type-2 tetrasaccharide, in agreement with previous findings that enteric bacteria lack the required enzyme specificity to catalyse the hydrolysis of the β1,3 linkage between LacNAc and Lac [48]. Although GH2 is a very common glycosidase present in intestinal bacteria, the presence of membrane bound β-galactosidases is limited across strains even across bifidobacteria [49]. All the β-galactosidase genes in *R. gnavus* E1 and ATCC 29149 are predicted to encode intracellular enzymes. The fact that, in the *R. gnavus* E1 genome, GH2 are often found clustered with CAZymes involved in plant degradation suggests that some of these enzymes may be involved in metabolism of plant substrates, in agreement with previous studies on transport and metabolism of plant cell wall oligosaccharides by *R. gnavus* E1 [42]. The lack of growth of *R. gnavus* strains on LNT is probably due to lack of an active GH20 lacto-N-biosidase; no GH20 is present in the ATCC 29149 genome and the two *R. gnavus* E1 GH20 enzymes (RUGNEv3_30022 and RUGNEv3_30140) show very little identity with functionally characterized GH20 lacto-N-biosidase from *Bifidobacterium bifidum* JCM1254 [47]. These predictions are further supported by the fact that *R. gnavus* E1 does not grow on LNT but grows on Lac, which indicates that *R. gnavus* E1 lacks lacto-N-biosidase specificity to cleave LNT into LNB and Lac.

The *R. gnavus* strains did not grow on 6′-sialyllactose (Neu5ACα2-6Galβ1-4Glc, 6′SL) but the ATCC 29149 strain grew well on 3′-sialyllactose (Neu5ACα2-3Galβ1-4Glc, 3′SL) (Fig. 2). The lack of *R. gnavus* E1 growth on these substrates is consistent with the absence of a GH33 encoding gene in the genome while it is present in the ATCC 29149 strain (Fig. 1). These results suggest that *R. gnavus* ATCC 29149 GH33 sialidase is specific for the α2,3- rather than α2,6-linkages. However since *R. gnavus* ATCC 29149 is unable to grow using either with Lac or sialic acid (Neu5Ac or Neu5Gc) as a sole source of carbon, the growth of *R. gnavus* ATCC 29149 on 3′SL was not expected (see below).

**Figure 4:**
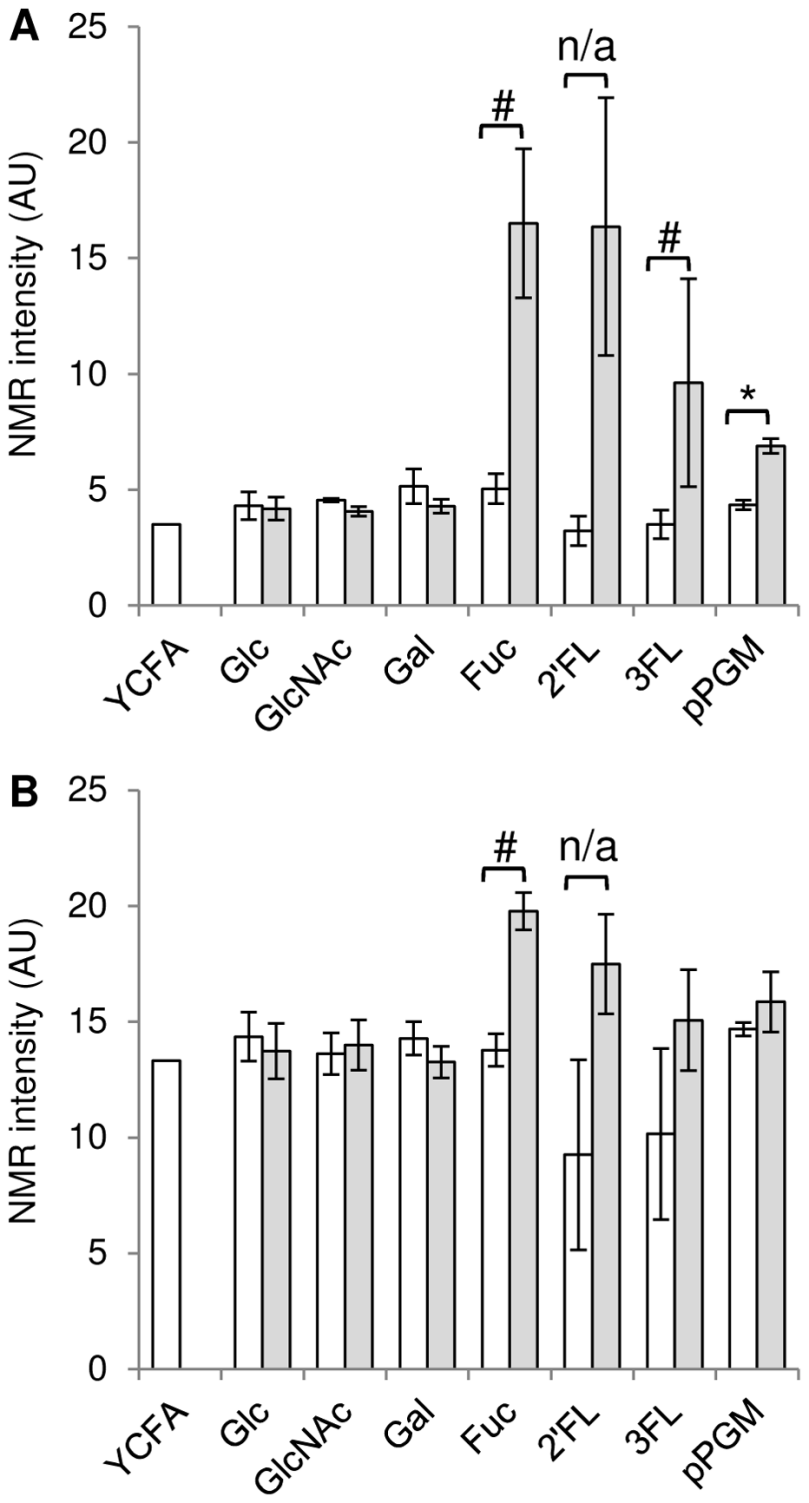
Quantification of propanol and propionate produced by *R. gnavus* ATCC 29149. The amount of propanol (A) and propionate (B) in the YCFA medium supplemented with different sugars has been quantified by ^1^H NMR before (control, white box) and after (grey box) growth of *R. gnavus* ATCC 29149. At least 3 replicates have been performed in each condition (except YCFA+2′FL control). For each sugar (except for 2′FL where there were insufficient number of replicates), a Mann-Whitney test was performed to compare the concentration of propanol or propionate in the medium before and after *R. gnavus* ATCC 29149 growth. Only the production of propanol by *R. gnavus* ATCC 29149 grown on pPGM was significant (*, p<0.05) but *R. gnavus* ATCC 29149 also seemed to produce both propanol and propionate when grown with Fuc as sole carbon source, and propanol when grown with 3FL as sole carbon source (#, p = 0.06). n/a: Not applicable.

Previous work has reported that *R. gnavus* was well adapted to mucin-degradation [50], [51], [52]. We grew *R. gnavus* ATCC 29149 and E1 strains in purified porcine gastric mucin (pPGM) to elucidate its competence in mucin degradation and utilisation. pPGM is a heavily glycosylated protein containing approximately 9.1% Fuc, 5.4% mannose (Man), 34% Gal, 28.9% GlcNAc, and 22.4% GalNAc in the *N*-glycans and 9.8% Fuc, 17.4% Gal, 32.3% GlcNAc, and 39.7% GalNAc in the *O*-glycans as determined by GC-MS and 1% (wt-%) sialic acids [32]. Despite its proficiency at using mucin-oligosaccharides (Gal, Fuc, GlcNAc) as carbon source, *R. gnavus* E1 failed to grow on mucin as sole carbon source, highlighting the importance of specific GHs in breaking up mucin complex carbohydrate chains to release assimilable oligosaccharides. In contrast, *R. gnavus* ATCC 29149 showed the ability to utilise mucin as source of carbon although to a lower density compared to oligosaccharides. While ATCC 29149 grew exponentially with almost no lag period on most oligosaccharides tested, a 1.5 h-lag period was observed in mucin-supplemented medium (Table 1, Fig. 2). ^1^H NMR analysis showed that there was a clear decrease in Fuc bound to mucin in the presence of *R. gnavus* ATCC 29149, suggesting that extracellular fucosidase activity plays an important role in the ability of this strain to grow on mucins (Fig. 3D). The ability of *R. gnavus* ATCC 29149 to utilise Fuc from fucosylated sources is in agreement with the metabolite analysis of *R. gnavus* supernatants, showing increasing propanol and propionate production (assumed to be via the propanediol pathway, [53]) when the bacteria are grown in presence of 3FL, 2′FL, Fuc and pPGM (Fig. 4, Fig. S1).

In order to further characterize the mechanisms by which *R. gnavus* ATCC 29149 grows on mucins, the supernatants of both *R. gnavus* strains grown on Glc and sialylated sources, 3′SL and mucin, were tested for sialidase activity using the synthetic substrate, 2′-(4-Methylumbelliferyl)-α-D-*N*-acetylneuraminic acid (4-MU-Neu5Ac). Sialidase activity (as measured by fluorescent assay) was detected in the spent media of *R. gnavus* ATCC 29149 grown in presence of 3′SL and mucin as compared to Glc (Table 2), whereas no sialidase activity was detected in the control experiment (without *R. gnavus* ATCC 29149) (data not shown), demonstrating that an active extracellular GH33 sialidase is produced by *R. gnavus* ATCC 29149.

**Table 2:** Enzymatic activity of *R. gnavus* ATCC 29149 supernatant grown on mucin and 3′SL on substrate 4-MU-Neu5Ac.

Culture time (h)	Glc	pPGM	3′SL
6	233±37	ND	898±64
9	302±13	1296±45	479±14
28	358±22	2160±87	613±22

ND-not detected, the rate of MU release was less than the negative control. Abbreviations; 3′SL-3′-sialyllactose, 4-MU-Neu5Ac-2′-(4-Methylumbelliferyl)-α-D-*N*-acetylneuraminic acid, MU-methylumbelliferone.

### *R. gnavus* ATCC 29149 transcriptomics reveal the importance of a functional nan gene cluster in mucin utilisation

To examine the molecular basis underlying host glycan utilisation of *R. gnavus* ATCC 29149, we then compared the CAZome transcriptome of *R. gnavus* ATCC 29149 grown on mucin, mucin glycans and HMOs. We used Custom Oligonucleotide Microarrays representing all predicted ORFs encoding CAZymes. Four probes per gene were designed for 96 of 98 CAZyme genes (see Protocol S1 for details) and were printed in duplicate on the array. The specific transcriptional response to growth on a particular glycan was determined after normalization using the signal obtained with genomic DNA hybridization. The level of expression was then compared to a reference dataset of the strain grown in minimal medium with Glc as the sole carbon source (Fig. S2). A distinct set of GHs were upregulated when *R. gnavus* ATCC 29149 consumed mucins and fucosylated glycans. GH29 (RUMGNA_03411) and GH95 (RUMGNA_00842) were specifically upregulated when grown on 2′FL and 3FL. GH29 RUMGNA_03411 and GH95 RUMGNA_00842 α-L-fucosidases possess an N-terminal signal sequence and a C-terminal LPxTG-like motif, suggesting that they act as extracellular membrane-bound enzymes. Another GH95 α-L-fucosidase, RUMGNA_03121, was preferentially upregulated when *R. gnavus* ATCC 29149 was grown in 3FL supplemented medium, although there is no predicted signal sequence. The GH33 sialidase (RUMGNA_02694) was specifically upregulated in presence of mucins, in agreement with the implication of this extracellular enzyme in enabling *R. gnavus* ATCC 29149 to grow on mucin (see above). Other mucin-specific upregulated genes include a predicted GH2 β-galactosidase (RUMGNA_01638) and a putative GH36 α-galactosidase (RUMGNA_03611), although both seem to be intracellular enzymes because of the lack of an N-terminal signal sequence.

**Figure 5:**
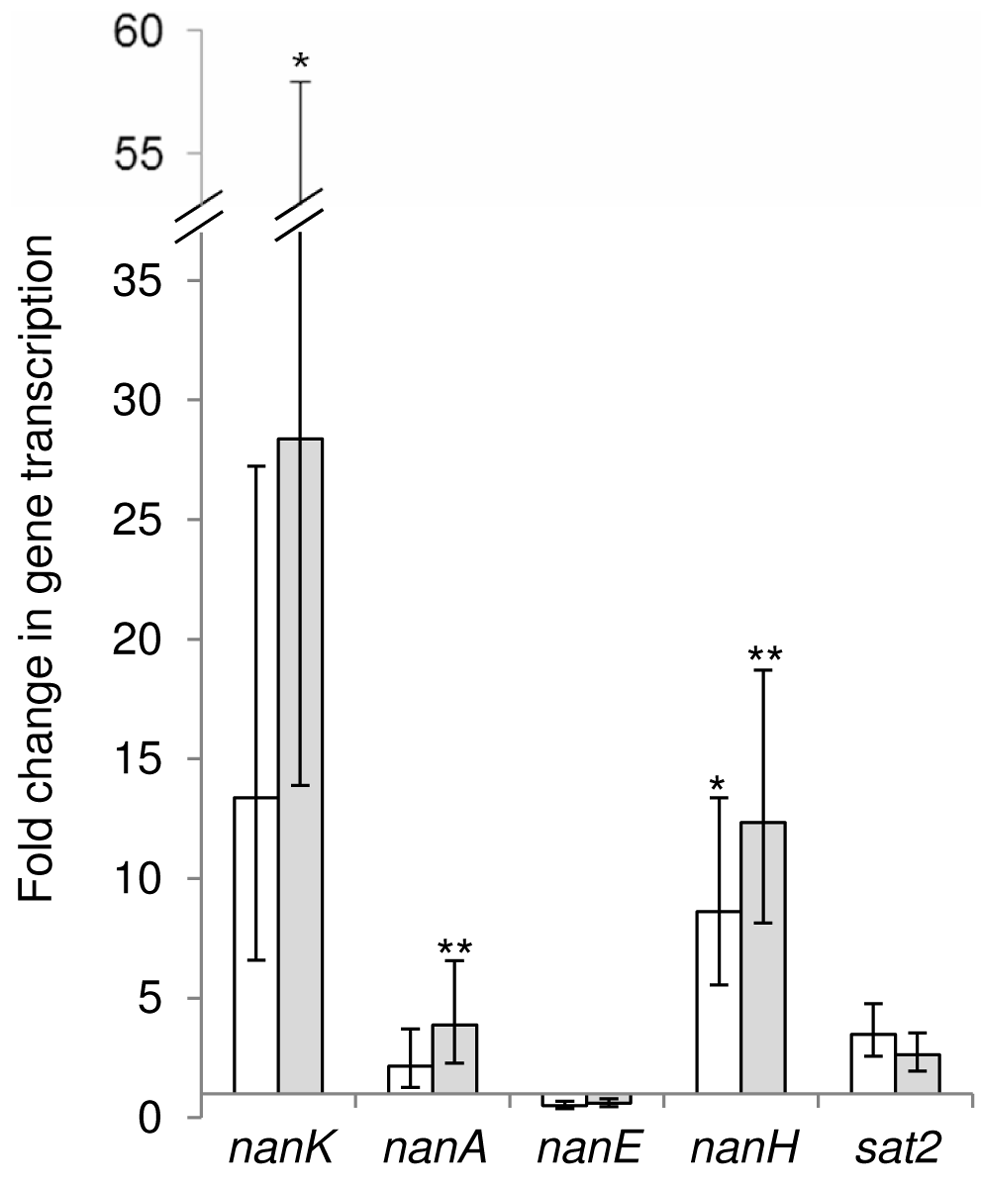
Relative level of transcription of *R. gnavus* ATCC 29149 *nan* genes. Fold change in gene transcription has been determined by qRT-PCR for the *nan* genes when *R. gnavus* ATCC 29149 was grown in presence of pPGM (white box) or 3′SL (grey box) compared to Glc as sole carbon source. The results showed averages of two biological replicates, each performed in 3 technical replicates. Data were analysed using 1-way ANOVA. For each gene, a post-hoc test (Dunnett's) was used to examine if there were any significant differences in each condition (versus Glc). The transcription of *nanH* was significantly increased when *R. gnavus* ATCC 29149 was grown with either pPGM or 3′SL compared to Glc. The transcription of both *nanK* and *nanA* was also significantly increased when ATCC 29149 was grown with 3′SL compared to Glc. *: p<0.05; **, p<0.01.

**Figure 6:**
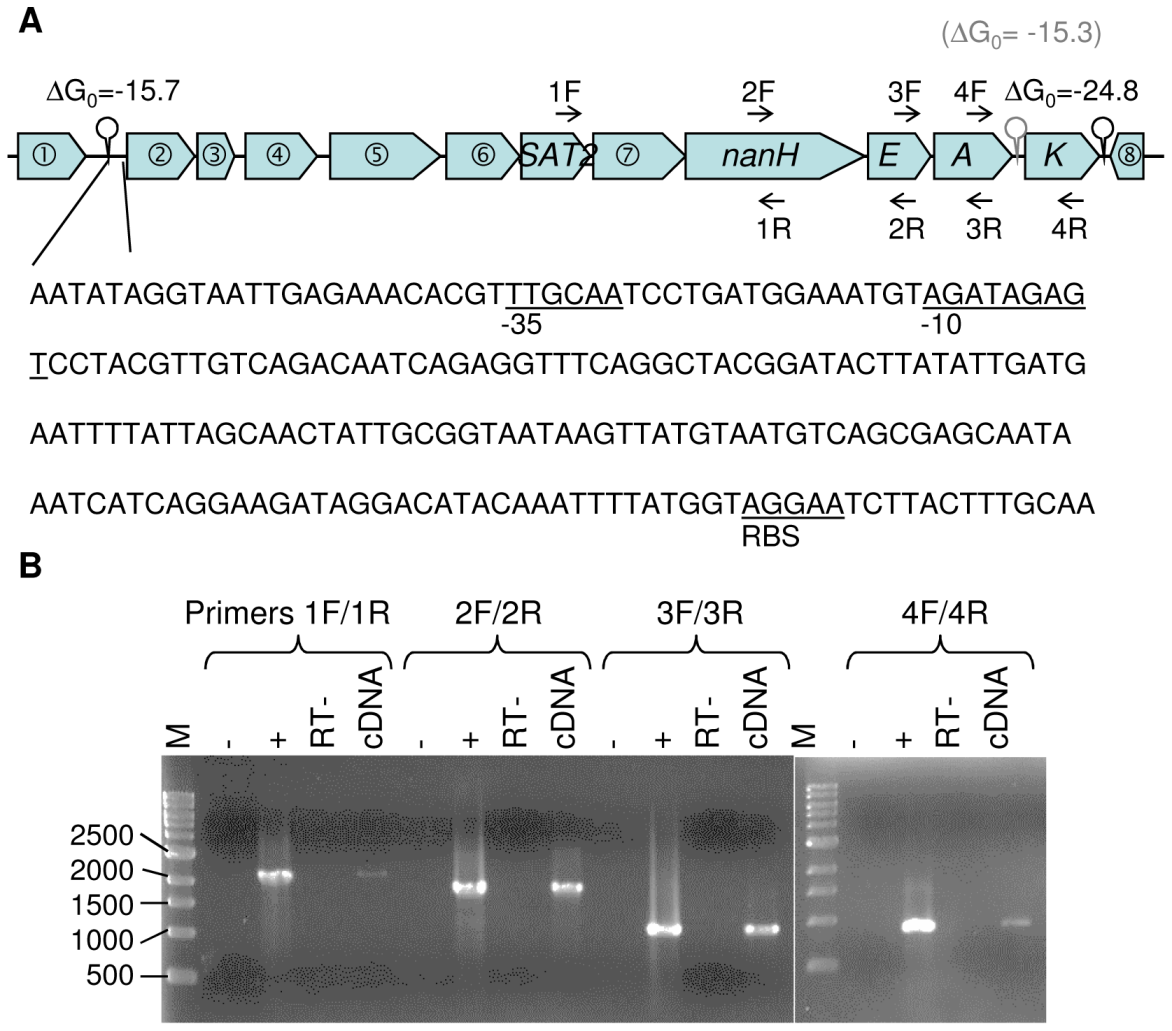
The *nan* locus in *R. gnavus* ATCC 29149. (A) Schematic representation of the *nan* genetic organization. Each block arrow indicates an ORF; the length of the arrow is proportional to the length of the predicted ORF. RUMGNA_02702, 02701, 02700, 02699, 02698, 02697, 02695 and 02690 are shown in block arrow 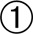 to 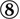, respectively. Circles above thick vertical lines indicate potential stem-loop structures that might act as Rho-independent transcriptional terminators. The free energy of the thermodynamic ensemble is given on top, expressed as kcal.mol^−1^. The inset shows the DNA sequence of the promoter located upstream of the putative RUMGNA_02701 gene (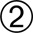). The putative −35 and −10 regions and ribosome-binding site (RBS) are underlined. (B) Confirmation of the *nan* operonic structure. The PCR products obtained following RT-PCR of RNA extracted from *R. gnavus* ATCC 29149 grown on pPGM were obtained using primers set spanning the SAT2 to NanK ORFs and analysed by electrophoresis on agarose gel. PCR from RT negative control (RT−) was performed to confirm the absence of genomic DNA contamination of the RNA sample prior to RT. PCR negative (−) and positive (+) controls were carried out with water or ATCC 29149 genomic DNA as template, respectively. The positions of the primers are shown in panel A and their sequences are provided in Table S1. M, DNA ladder size marker (with increments indicated in base pairs).

qRT-PCR analysis was performed on RNA extracted from *R. gnavus* ATCC 29149 grown on different sugars. The data were normalized using *gyrB* (RUMGNA_00867) as a reference gene and expressed as a fold change in gene expression compared to Glc. These experiments revealed the physiological significance of the *nan* cluster in mucin metabolism (Fig. 5). This gene cluster contains 11 open reading frames (ORFs) (Fig. 6). The first gene of the cluster encodes a protein of unknown function. The second gene (RUMGNA_02700) encodes a putative sugar isomerase involved in sialic acid catabolism. The following one (RUMGNA_02699) encodes a protein with homology with transcriptional regulators of the AraC family. The following 3 genes code for a predicted solute-binding protein (RUMGNA_02698) and two putative permeases (RUMGNA_02697, RUMGNA_02696), components of a sugar ABC transporter; RUMGNA_02696gp has specific homology with putative sialic acid transporters of the SAT2 family [54]. The following gene has no known function. The sialidase gene *nanH* (RUMGNA_02694) predicted to encode the GH33 enzyme comes next. Then *nanE* (RUMGNA_02693), which encodes a predicted ManNAc-6-P epimerase converting ManNAc-6-P into *N*-acetylglucosamine-6-P (GlcNAc-6-P) followed by *nanA* (RUMGNA_02692) encoding a putative Neu5Ac lyase involved in the breaking down of Neu5Ac into *N*-acetylmannosamine (ManNAc) and phosphoenolpyruvate (PEP). *nanK* (RUMGNA_02691) is the last gene of the cluster, coding for a predicted ManNAc kinase. This 11.7-kb region thus contains genes that appear to be involved in the metabolism and transport of sialic acid (Fig. 6A). Indeed, almost all the genes putatively involved in sialic acid utilization (*nan* genes) as well as the potential SAT2 transporter RUMGNA_02696gp were upregulated when the bacterium was grown with mucin as sole carbon source. The qRT-PCR also confirmed induction of RUMGNA_02694 coding for a GH33 sialidase as shown by *R. gnavus* ATCC 29149 CAZyme microarray analyses. Only the *nanE* gene (RUMGNA_02693) was not upregulated but high level of expression was already present when *R. gnavus* ATCC 29149 was grown in Glc (Fig. 5). Transcriptional terminator prediction suggests that the 10 genes from RUMGNA_02701 to *nanA* form part of a single operon.

To confirm this bioinformatics analysis, RT-PCR analysis using primer sets encompassing the neighboring ORFs (RUMGNA_02696 to RUMGNA_02691) was performed on total RNA extracted from a mid-logarithmic phase culture of *R. gnavus* ATCC 29149 grown with mucins or 3′SL as sole carbon source. The data showed that genes encoding the potential SAT2 transporter RUMGNA_02696gp, GH33 sialidase (RUMGNA_02694gp), NanE (RUMGNA_02693gp) and NanA (RUMGNA_02692gp) were co-transcribed. Interestingly, *nanK* (RUMGNA_02691) also seemed to be co-transcribed with *nanA* while a transcriptional terminator was predicted between the two genes (Fig. 6B). Taking together, our data suggest that the 11 genes of the cluster are organized in an operon, which is transcribed from the promoter upstream of the RUMGNA_02701 gene.

**Figure 7:**
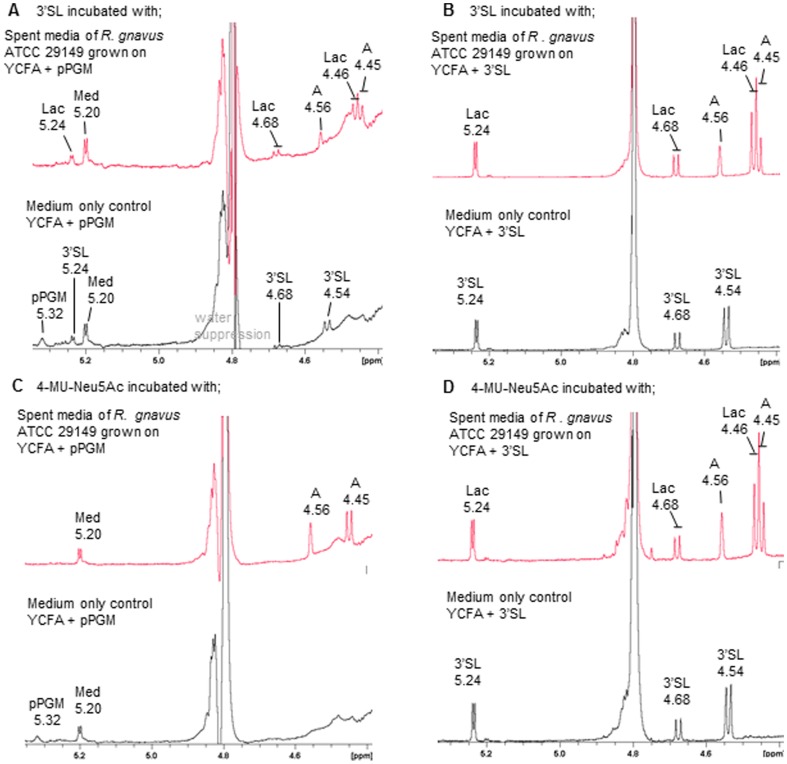
^1^H NMR analysis of sialylated substrates incubated with spent media of *R. gnavus* ATCC 29149. *R. gnavus* ATCC 29149 was grown in YCFA supplemented with pPGM or 3′SL for 9 h and the cells removed by centrifugation. 3′SL was incubated with spent media supplemented with pPGM (A) and 3′SL (B). 4-MU-Neu5Ac was incubated with spent media supplemented with pPGM (C) and 3′SL (D). The control media without inoculation with *R. gnavus* are shown in the lower trace of each panel. Abbreviations; 3′SL-3′-sialyllactose, Lac-lactose, A- 2,7-anhydro-Neu5Ac, pPGM-purified porcine gastric mucin, med-unidentified media component.

The presence of a complete *nan* cluster (*nanE*, *nanA*, *nanK*), and potential GH33-coding *nanH* and SAT2 transporter-coding RUMGNA_02696 in *R. gnavus* ATCC 29149 operon, together with their increased expression in response to mucins and 3′SL, suggest that this strain has adapted to scavenge sialic acid from sialylated substrates. However this is in disagreement with the lack of *R. gnavus* ATCC 29149 growth in presence of sialic acid as sole carbon source (see above). In order to further investigate the underpinning mechanisms of ATCC 29149 growth on a sialylated carbon source, the supernatant of *R. gnavus* ATCC 29149 grown on mucin or 3′SL and shown to produce an active sialidase (see above), was used in an *in vitro* assay in presence of 4-MU-Neu5Ac or 3′SL as substrate and the products of the reaction monitored by ^1^H NMR (Fig. 7). The spectra clearly showed the presence of peaks identified as 2,7-anydro-α-N-actetylneuraminic acid (2,7-anhydro Neu5Ac) [55], [56] when *R. gnavus* ATCC 29149 grown on mucin or 3′SL was used as a “source of sialidase” (Fig. 7 A/B). The signals of 2,7-anhydro Neu5Ac and their chemical shifts are shown in Table S2. This product was absent in control experiments using supernatant containing 3′SL or mucin in absence of *R. gnavus* ATCC 29149 (Fig. 7 C/D), confirming the specificity of the enzymatic reaction.

## Discussion

Most gut bacteria species belong to the phyla Firmicutes, Bacteroidetes, Actinobacteria, Proteobacteria and Verrucomicrobia but only a few members have been studied for their ability to degrade mucins [57]. This is in particular the case of the Gram-negative human gut symbiont, *Bacteroides thetaiotaomicron* which, in the absence of dietary nutrients, relies on host-derived glycans (mucins) for colonization [58]. Genome analysis of Bacteroides revealed a subset of polysaccharide utilization loci (PULs) dedicated to host mucin *O*-glycans [59], [60]. Within the Actinobacteria phylum, detailed genome analysis of Bifidobacteria identified metabolic pathways for the degradation of mucin-type *O*-glycan and HMOs and several GHs have been functionally characterized supporting these findings [61]. Recently, another constituent of the human gut microbiota, *Akkermansia muciniphila*, a strictly anaerobic Gram-negative bacterial species, was identified as an important mucin-degrader of the Verrucomicrobia phylum [62]. In sharp contrast, the mucin glycan acquisition strategies of Firmicutes, which are prominent members of the human microbiota, remain ill-defined.

The Gram-positive *R. gnavus* belongs to the *C. coccoides* group within the Firmicutes phylum. On average, sequenced Firmicutes encode fewer CAZymes than Bacteroidetes but possess more ABC transporters that transport carbohydrates [4]. Although both *R. gnavus* strains under study dedicate a similar percentage of their genome to CAZymes (∼2.5–3.7%), a close inspection of their CAZomes, highlighted differences in specific GH families. The capacity of *R. gnavus* ATCC 29149 and not *R. gnavus* E1 to utilise mucins suggests that the difference in mucin-utilization pathways is most likely due to the expression of specific GH extracellular enzymes in ATCC 29149.

In mucins, fucosyl residues can be found at the extremity of the *O*-glycosidic chain linked to galactose by α-1,2 linkage or to GlcNAc by α-1,3 linkage whereas it is most commonly linked α-1,6 to the reducing terminal β-GlcNAc in human *N*-linked glycans. Since Fuc was shown to be a good substrate for both *R. gnavus* ATCC 29149 and E1, and both strains possess a great number of fucosidase-encoding genes, the growth difference between the two strains on mucin may be due to the substrate specificity of the *R. gnavus* ATCC 29149 enzymes for the release of Fuc from pPGM. Genome analysis showed that *R. gnavus* ATCC 29149 encodes two putative GH29 (RUMGNA_03411 and RUMGNA_03833) and three putative GH95 α-L-fucosidases (RUMGNA_00842, RUMGNA_01058 and RUMGNA_03121). Among these, RUMGNA_03411 and RUMGNA_00842, are upregulated in presence of 2′FL and 3FL and and both predicted to be anchored to the cell wall. Furthermore GH95 RUMGNA_00842 and GH29 RUMGNA_03411 show around 62.5% and 55.5% homology to *Bifidobacterium bifidum* JCM1254 GH95 AfcA specific for the α1,2-linkage [63] and GH29 AfcB specific for the α1,3- and α1,4-linkages [64], which can remove Fuc at the non-reducing termini except for any that are α1,6-linked. Furthermore AfcA catalytic residues are conserved in GH95 RUMGNA_00842 and although AcfcB catalytic residues have not been functionally determined, RUMGNA_03411 has the conserved nucleophile in GH29 family (the general acid/base of GH29 cannot be unambiguously assigned by multiple alignments). Together these data suggest that RUMGNA_03411 and RUMGNA_00842 play a key role in the ability of *R. gnavus* ATCC 29149 to grow on mucins.

The release of sialic acids from non-reducing ends is an initial step of sequential degradation of mucins since sialic acid residues may prevent the action of other GHs. In bacteria, the genes involved in sialic acid metabolism are usually found clustered together forming what is denominated as the Nan cluster encoding the enzymes *N*-acetylneuraminate lyase (NanA), epimerase (NanE), and kinase (NanK), converting Neu5Ac into GlcNAc-6-P whereas the genes encoding NagA (GlcNAc-6-P deacetylase) and NagB (glucosamine-6-P deaminase) converting GlcNAc-6-P into fructose-6-P (Fru-6-P), which is a substrate in the glycolytic pathway, vary in their locations among the different genomes that encode the Nan cluster [65]. *R. gnavus* is one of the few human gut commensals that encode the Nan cluster along with *Anaerotruncus colihominis*, *Dorea formicigenerans*, *D. longicatena*, *F. prausnitzii*, *Fusobacterium nucleatum*, *Lactobacillus sakei*, *L. plantarum*, and *L. salivarius* [66]. The majority of the bacteria that encode the Nan cluster colonize mucus regions of the human body, such as the gut, lung, bladder or oral cavity, where sialic acid is highly abundant and can serve as a source of energy, carbon, and nitrogen [66]. However, prior to its catabolism, sialic acid has to be cleaved off from sialylated glycans by a GH33 sialidase (NanH) and transported into the cell. To date there are three functionally characterised sialic acid transporters: NanT, a single component system, a tripartite ATP-independent periplamic C4-dicarboxilate (TRAP) multicomponent transport system and an ATP-binding cassette (ABC) transporter (SAT). In addition four new putative types of sialic acid transporters were recently identified i.e. two other types of ABC-transporters (SAT2 and SAT3), a sodium-glucose/galactose cotransporter (SSS) and a Na+/proline symporter (Sym) [54]. There is a homologue to SAT2-type transporter next to the *R. gnavus* ATCC 29149 Nan cluster (RUMGNA_02696), sharing high level of homology (72% identity/86% similarity) with the putative sialic transporter from *Streptococcus sanguinis* SK36, suggesting that *R. gnavus* ATCC 29149 is well equipped to utilise Neu5Ac as carbon source. In addition the relative position of the Nan genes in *R. gnavus* ATCC 29149 is identical to *D. formicigenerans* ATCC 27755 and *D. longicatena* DSM 13814 and the one in *Clostridium perfringens* SM101, an opportunistic pathogen in the gut. However in these organisms, the transporter belongs to the SSS type and is located between NanA and NanK. Interestingly we showed that despite the presence of the Nan cluster and putative sialic acid transporter, *R. gnavus* was unable to utilize sialic acid as sole carbon source but selectively grew on α2-3 linked sialylated substrate and mucins, showing sialidase activity as assessed using synthetic fluorescent substrate (4MU-Neu5Ac), production of 2,7-anhydro-Neu5Ac *in vitro* and upregulation of Nan genes, putative GH33 sialidase and SAT2-type transporter *in vivo*. Taken together, our data suggest that *R. gnavus* ATCC 29149 encodes an intramolecular *trans*-sialidase (IT-sialidase) producing 2,7-anhydro-Neu5Ac selectively from α2-3 linked sialic acid substrates. This product may be transported into the bacteria by SAT2 and further metabolized into the cell by the enzymes encoded by the Nan cluster, supporting bacterial growth on 3′SL or mucin. To date only two enzymes with IT-sialidase activity have been reported, NanL from *Macrobdella decora* (North American leech) [67] and NanB from the human pathogen *Steptococcus pneumoniae* [68]. This is the first report of intramolecular transialidase activity in gut commensal bacteria, suggesting an unprecedented mechanism underpinning adaptation of gut bacteria to the mucosal environment.

## Conclusions

Our findings show that *R. gnavus* strains typically display a subset of glycan-degrading phenotypes that may equip them to target just part of the overall glycan repertoire present at certain times or locations of the gastrointestinal tract. The ability of *R. gnavus* ATCC 29149 to access the glycans attached to mucus may have a role in early colonization by providing some bacteria with a source of endogenous nutrients during a period when dietary glycans are absent. A recent study showed that *R. gnavus* was predominant in breast milk/goat milk-fed microbiotas compared to a more diverse collection of Lachnospiraceae in cow milk-fed babies [69]. In adults, the ability to metabolize the mucin *O*-linked oligosaccharides is likely to be a key factor in determining which microorganisms associate at the mucosal surface. Given the link between the microbiota and gut inflammatory processes, mucin-degraders may represent prime members influencing the host immune response. As such, our results suggest that bacterial IT-sialidases may play a key role in driving commensal and/or symbiotic host associations. Dissecting the molecular strategies used by *R. gnavus* strains to degrade and utilize mucin glycans is important for understanding the genetic and associated metabolic properties that underpin adaptation to the gut mucosal environment.

## Supporting Information

Figure S1 **^1^H NMR spectra of propanol and propionate production by** ***R. gnavus*** **ATCC 29149.** Culture supernatants of *R. gnavus* ATCC 29149 grown in presence of different sugars as sole carbon source were analysed by H1 NMR. These portions of the H1 NMR spectra show a substantial increase of the peaks from propanol at 1.53 ppm (A) and propionate at 2.17 ppm (B) when the strain was grown with Fuc or fucosylated substrates. Black: no sugar; light grey: Glc; Dark grey: GlcNAc; Dark blue: Gal; Light pink: Fuc; Pink: 2′FL; Purple: 3FL and Red: pPGM.
(TIF)

Figure S2 **Microarray data of all CAZyme genes clustered by family.** Transcriptomic analysis of all *R. gnavus* ATCC 29149 CAZyme genes has been performed by microarray in response to different carbon sources (Glc, Gal, Fuc, 2′FL, 3FL or pPGM). Details of the protocol regarding probe design, sample preparation, microarray hybridization and data analysis can be found in Material and Methods and in Protocol S1. The level of expression of the genes, clustered by family, is indicated by a color code from blue (low level of expression) to red (high level of expression). The shade of the color provides the level of trust based on the variability obtained with different probes for one gene.
(TIF)

Table S1 **Primers used for qRT-PCR and RT-PCR.**
(DOCX)

Table S2 **Signals of 2,7-anhydro Neu5Ac and their chemical shifts.**
(DOCX)

Protocol S1 **Transcriptional profiling by microarray.**
(DOCX)
