# Supplementary figures and images for: Utilisation of Mucin Glycans by the Human Gut Symbiont Ruminococcus gnavus Is Strain-Dependent
Source: PLoS One. 2013 Oct 25;8(10):e76341. doi: 10.1371/journal.pone.0076341 (PMC3808388; doi:10.1371/journal.pone.0076341)

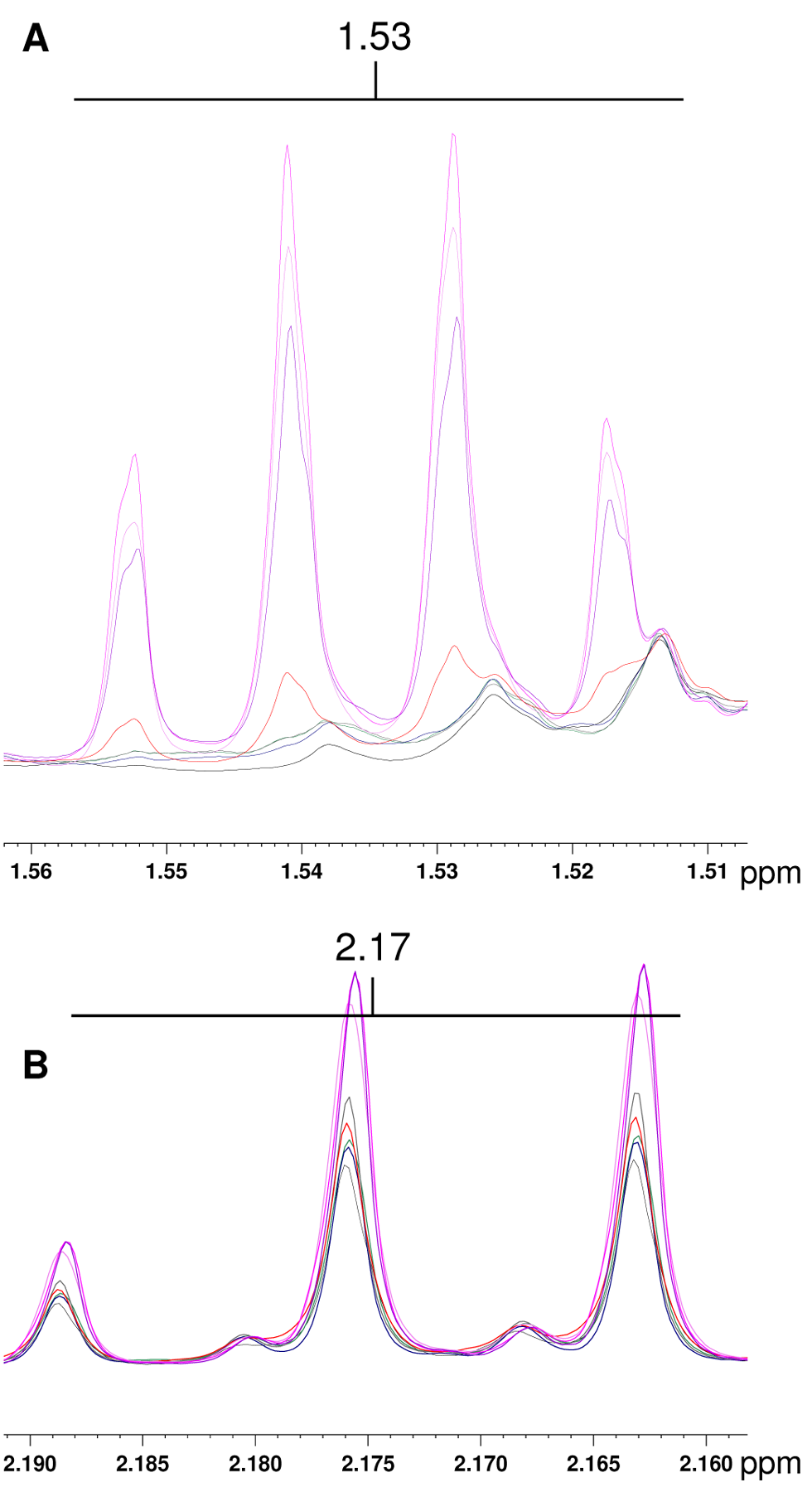

Supplement: Figure S1 — 1H NMR spectra of propanol and propionate production by R. gnavus ATCC 29149. Culture supernatants of R. gnavus ATCC 29149 grown in presence of different sugars as sole carbon source were analysed by H1 NMR. These portions of the H1 NMR spectra show a substantial increase of the peaks from propanol at 1.53 ppm (A) and propionate at 2.17 ppm (B) when the strain was grown with Fuc or fucosylated substrates. Black: no sugar; light grey: Glc; Dark grey: GlcNAc; Dark blue: Gal; Light pink: Fuc; Pink: 2′FL; Purple: 3FL and Red: pPGM. (TIF) [file pone.0076341.s001.tif]

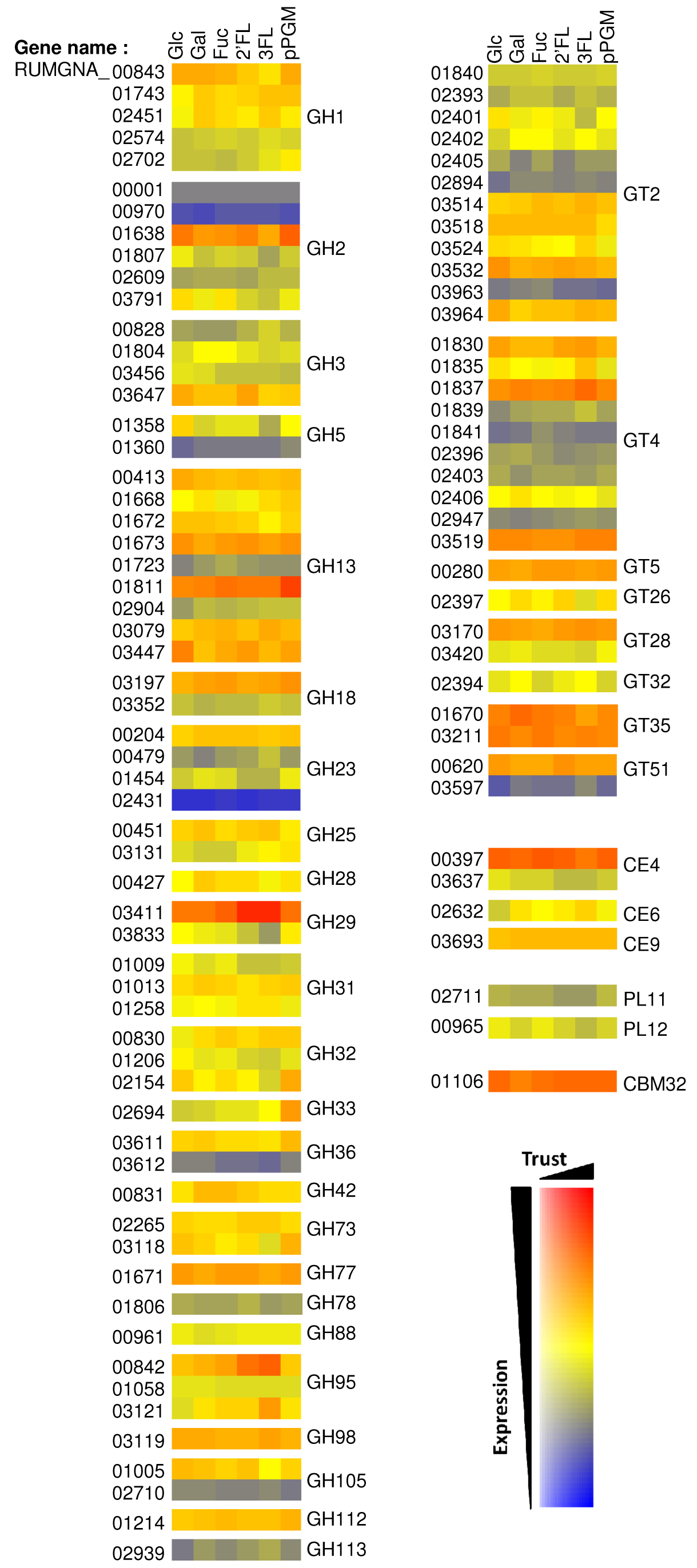

Supplement: Figure S2 — Microarray data of all CAZyme genes clustered by family. Transcriptomic analysis of all R. gnavus ATCC 29149 CAZyme genes has been performed by microarray in response to different carbon sources (Glc, Gal, Fuc, 2′FL, 3FL or pPGM). Details of the protocol regarding probe design, sample preparation, microarray hybridization and data analysis can be found in Material and Methods and in Protocol S1. The level of expression of the genes, clustered by family, is indicated by a color code from blue (low level of expression) to red (high level of expression). The shade of the color provides the level of trust based on the variability obtained with different probes for one gene. (TIF) [file pone.0076341.s002.tif]
