# Supplementary material for: Utilisation of Mucin Glycans by the Human Gut Symbiont Ruminococcus gnavus Is Strain-Dependent
Source: PLoS One. 2013 Oct 25;8(10):e76341. doi: 10.1371/journal.pone.0076341 (PMC3808388; doi:10.1371/journal.pone.0076341)
Supplement: Table S1 — Primers used for qRT-PCR and RT-PCR. (DOCX) [file pone.0076341.s003.docx]

**Table S1 Primers used for qRT-PCR and RT-PCR**

| Gene name | Forward primer | Reverse primer | amplicon size (nt) |
| --- | --- | --- | --- |
| RUMGNA_00867 (*gyrB*) | ggagcagaccagatccaaat | ccaatatacattcccggtcttt | 68 |
| RUMGNA_00842 | gggaagcgttgcagtagaac | caccttgatctcggttcctg | 61 |
| RUMGNA_01058 | atccggaaagaccagactcc | tttccagacgtcgatccaat | 67 |
| RUMGNA_01638 | ccacaggttcttatgtccgttt | atcaccctttttccgatcaa | 62 |
| RUMGNA_01811 | ttatcatgtggacgggcttc | tcctgctttccatagtccaga | 69 |
| RUMGNA_02691 | tgggatggataccatatcttcg | caatggcacgattacaaatca (4R) | 64 |
| RUMGNA_02692 | ctggaacttggtgcagtaagag (4F) | aatctccatgtcctcatctacca (3R) | 60 |
| RUMGNA_02693 | tgcaggagtcaaacacaagg (3F) | ccttgccttttggggtgta (2R) | 61 |
| RUMGNA_02694 | atagactggccacgggatt (2F) | tgtaggaagcactcccttgtatc (1R) | 61 |
| RUMGNA_02696 | ggtttccgtggcattaaaatc (1F) | ccatcatatctccccagttca | 62 |
| RUMGNA_03119 | ctgagagtcgtatctccgaagg | atctactgacggaaggaaacga | 60 |
| RUMGNA_03121 | gggaacagagattacggtgaa | aaccactgggcaaactcatatc | 60 |
| RUMGNA_03411 | aatcacacctggggatgatatt | ccttctacggaagcattcaca | 60 |
| RUMGNA_03611 | ttcggaaaacgggaggat | gcttttccagattcggatacc | 61 |
| RUMGNA_03829 | gaagtaggtgcaaacccatgta | agatccttctgccagtgcat | 60 |
| RUMGNA_03833 | ccaattacggaaagctggat | tctgctttccatgtatcttcaca | 76 |
