# Supplementary material for: Utilisation of Mucin Glycans by the Human Gut Symbiont Ruminococcus gnavus Is Strain-Dependent
Source: PLoS One. 2013 Oct 25;8(10):e76341. doi: 10.1371/journal.pone.0076341 (PMC3808388; doi:10.1371/journal.pone.0076341)
Supplement: Table S2 — Signals of 2,7-anhydro Neu5Ac and their chemical shifts. (DOCX) [file pone.0076341.s004.docx]

**Table S2** **Signals of 2,7-anhydro Neu5Ac and their chemical shifts**

|  | Chemical shift (ppm) | | | | | | |
| --- | --- | --- | --- | --- | --- | --- | --- |
|  | H-3ax | H-3eq | H-4 | H-5 | H-6 | H-7 | 5-Ac |
| H | 2.18 | 2.02 | 4.09 | 3.94 | 4.56 | 4.45 | 2.05 |
| C | 35.44 | 35.44 | 67.85 | 52.16 | 77.01 | 76.63 | 21.93 |
