# Supplementary material for: Utilisation of Mucin Glycans by the Human Gut Symbiont Ruminococcus gnavus Is Strain-Dependent
Source: PLoS One. 2013 Oct 25;8(10):e76341. doi: 10.1371/journal.pone.0076341 (PMC3808388; doi:10.1371/journal.pone.0076341)
Supplement: Protocol S1 — Transcriptional profiling by microarray. (DOCX) [file pone.0076341.s005.docx]

**Protocol S1**

**Transcriptional profiling by microarray**

Probe and microarray design:

A total of 1499 60-mer probes were designed for microarray experiments based on *R. gnavus* ATCC 29149 genome information using Array Designer 3.0 software (PREMIER Biosoft International, Palo Alto, CA). Among these, 387 probes were specific for the 98 CAZyme genes: 3 probes for the gene RUMGNA_03963, no probe for RUMGNA_00001 and 4 probes per gene for the other 96 CAZyme genes. All these probes were printed in duplicate on Agilent Custom Oligonucleotide Microarrays 8 x 15k. The other 1112 probes were designed based on sequence information of 577 randomly picked non-CAZyme genes: 2 probes per gene for 535 genes and one probe per gene for 42 genes. Only 26 of these probes could be printed in duplicate on the Agilent microarray. To summarize, a total of 1912 probes specific for *R. gnavus* ATCC 29149 genes were printed on each array. Each array also contained 150 replicative probes (15 probes printed 10 times each) and 536 Agilent control probes for QA purpose.

Sample preparation:

ATCC 29149 genomic DNA (reference gDNA, 25 μg) were digested by Sau3AI and the reaction mix purified by phenol-chloroform-isoamyl alcohol (25:24:1) and chloroform-isoamyl alcohol (24:1) treatment using MaXtract High Density tubes (Qiagen) according to supplier’s recommendations. The DNA was then precipitated with 0.3 M sodium acetate (pH5.2) and 70% ice-cold ethanol. After drying, the DNA pellet was resuspended in 50 μL TE buffer.

DNAse-treated RNA (10 μg) were converted into cDNA using random primers (Life Technologies Ltd) and AffinityScript Multiple Temperature Reverse Transcriptase (Agilent Technologies) following the manufacturer’s recommendation except for the incubation at 42°C which was extended overnight. Denaturation of the reverse-transcriptase and the RNA matrix was then performed by addition of 43 mM sodium hydroxide followed by a 10 min-incubation at 70°C. The reaction mixture was neutralized with 30 mM hydrochloric acid and then cleaned up using DyeEx® 2.0 Spin Kit (Qiagen) according to supplier’s recommendation.

The digested gDNA and each cDNA (6 μg) were then fluorescently labeled using the BioPrime® Array CGH Genomic Labeling System (Life Technologies Ltd) according to supplier’s instructions, and Cy3-dUTP or Cy5-dUTP respectively (GE Healthcare UK Ltd, Little Chalfont, UK). The reaction product was purified using DyeEx® 2.0 Spin Kit (Qiagen) according to manufacturer’s recommendation. The quantity of Cy3-labeled gDNA and Cy5-labeled cDNA, and the degree of labeling were assessed by NanoDrop 1000 UV-Vis Spectrophotometer.

Microarray:

The Cy5-cDNA (2 μg) was mixed with 1 μg of Cy3-gDNA, dried using the Savant SpeedVac™ SC110 (Thermo Fisher Scientific) and resuspended in 20 μL sterile ultrapure water. Then, 25 μL of 2x Hi-RPM Hybridization Buffer and 5 µL of 10x Gene Expression Blocking Agent (Agilent Technologies) were added, and the reaction mixture boiled for 3 min. Eight different Cy5-cDNA/Cy3-gDNA mixtures were prepared as above. The microarrays were hybridized overnight at 63°C with 40 μL of reaction mixture. After hybridization, the slide was washed and dried according to Agilent’s protocol. The slides were scanned on GenePix® 4000B scanner (Molecular Devices, Inc., Sunnyvale, CA) at 532 and 635 nm using 5-μm resolution. Image processing was done with GenePix Pro 6.0 software (Molecular Devices, Inc.). Foreground and local background intensities of each spot were characterized by both the mean and median pixel values of the spot for both dye channels. Data analysis was then performed using background-corrected median signal intensities from each dye channel using GeneSpringGX version 7.3 software (Agilent Technologies); A per spot and per chip intensity-dependent normalization (also called LOWESS normalization) was applied using corrected signal obtained for Cy3-gDNA at 532 nm as a control signal.
